# Postrecurrence Treatment in Neoadjuvant or Adjuvant FDA Registration Trials: A Systematic Review

**DOI:** 10.1001/jamaoncol.2024.1569

**Published:** 2024-06-20

**Authors:** Timothée Olivier, Alyson Haslam, Vinay Prasad

**Affiliations:** 1 Department of Oncology, Geneva University Hospital, Geneva, Switzerland; 2 Department of Epidemiology and Biostatistics, University of California, San Francisco

## Abstract

**Importance:**

In oncology randomized clinical trials, suboptimal access to best available care at recurrence (or relapse) may affect overall survival results.

**Objective:**

To assess the proportion and the quality of postrecurrence treatment received by patients enrolled in US Food and Drug Administration (FDA) registration trials of systemic therapy in the adjuvant or neoadjuvant setting.

**Evidence Review:**

For this systematic review, all trials leading to an FDA approval from January 2018 through May 2023 were obtained from the FDA website and drug announcements. Randomized clinical trials of an anticancer drug in the neoadjuvant or the adjuvant setting were included. Trials of supportive care treatment and treatments given in combination with radiotherapy were excluded. Information abstracted for each trial included tumor type, setting, phase, type of sponsor, reporting and assessment of postrecurrence, and overall survival data.

**Findings:**

A total of 14 FDA trials met the inclusion criteria. Postrecurrence data were not available in 6 of 14 registration trials (43%). Of the 8 remaining trials, postrecurrence treatment was assessed as suboptimal in 6 (75%). Overall, only 2 of 14 trials (14%) had data assessed as appropriate.

**Conclusions and Relevance:**

This systematic review found that 43% of randomized clinical trials of anticancer treatment in the adjuvant or neoadjuvant context failed to present any assessable postrecurrence treatment data. In instances in which these data were shared, postrecurrence treatment was suboptimal 75% of the time. The findings suggest that regulatory bodies should enforce rules stipulating that patients have access to the best standard of care at recurrence.

## Key Points

**Question:**

How often is postrecurrence treatment reported in adjuvant and neoadjuvant oncology randomized clinical trials, and what is the access to optimal postrecurrence treatment?

**Findings:**

In this systematic review of 14 US Food and Drug Administration registration trials of systemic therapy in the neoadjuvant or adjuvant setting from 2018 to 2023, postrecurrence treatment was not reported in 43% of trials. Overall, 14% of trials had data assessed as appropriate.

**Meaning:**

The findings suggest that regulatory rules should enforce stricter requirements regarding postrecurrence treatment access and reporting in trials.

## Teaser

This systematic review examines the proportion and quality of postrecurrence treatment among patients in US Food and Drug Administration (FDA) registration trials of anticancer therapy in the neoadjuvant or adjuvant setting.

## Introduction

In patients with cancer undergoing curative local treatment, neoadjuvant and adjuvant systemic therapies aim to lower recurrences, increase cure rates, or permit less invasive surgery. Increasing cure rate applies to the whole population because some patients benefit from additional therapy while others do not, and many experience adverse effects. Patients who benefit are a minority of treated patients.^1^

All drugs used in the adjuvant context could also be prescribed in metastatic settings.^2^ Therefore, the question in adjuvant setting is whether to treat all patients earlier despite many not benefiting or to only treat at recurrence. A valid answer is obtained if patients who have recurrence in the control arm have optimal access to therapeutic options, including drugs tested in the neoadjuvant or adjuvant setting when indicated. We aimed to evaluate the reporting and characteristics of postrecurrence therapy in trials leading to US Food and Drug Administration (FDA) registration of systemic therapy in the adjuvant or neoadjuvant treatment setting.

## Methods

In this systematic review, we sought to identify all randomized clinical trials of anticancer treatment in the adjuvant or neoadjuvant setting leading to a marketing authorization (from January 2018 through May 2023). Searches of the FDA website and drug announcements were performed on June 10, 2023.^3^ Trials of supportive treatments and pediatric trials were excluded, as were trials of therapies given in combination with radiotherapy. We abstracted trial, postrecurrence, and overall survival (OS) data.

We evaluated postrecurrence data using prespecified rules with the same principles as in a previous work.^4^ The first rule assessed the type of therapy that patients received at disease recurrence and was met when less than 10% of patients receiving systemic therapy at disease recurrence were deprived from a therapy already proven to be a preferred beneficial option, considered the standard of care (eMethods in Supplement 1). The second rule assessed crossover and was satisfied when no more than 10% of control patients receiving systemic treatment after relapse received the experimental drug if the drug’s efficacy in later stages was not yet proven. The third rule assessed the overall access to any therapy at recurrence. This rule was fulfilled when the number of patients receiving any treatment after disease recurrence was at least 10% higher in trials than outside trials (eResults 2 in Supplement 1). Since first recurrence may be amenable to local treatment, we did not restrict the assessment of the third rule to systemic therapy only. Additional details are described in the eMethods in Supplement 1.

### Statistical Analysis

Frequencies were calculated for categorical variables. R, version 4.1.2 (R Foundation) was used for statistical analysis.

## Results

Of 272 FDA approvals, 14 trials^5,6,7,8,9,10,11,12,13,14,15,16,17,18^ met the inclusion criteria (eFigure 1 in Supplement 1). All trials were industry-sponsored phase 3 trials leading to regular approvals. Other trial characteristics are described in Table 1.

**Table 1.** Registration Trials Leading to an FDA Approval in the Neoadjuvant or Adjuvant Setting Between 2018 and 2023

Trial name	Experimental arm^a^	Mechanism of action (target)	Control	Design	Phase	Date of FDA approval	Tumor type	End points	
								Primary	Selected secondary
COMBI-AD^6^	Dabrafenib-trametinib	Kinase inhibitor (BRAF, MEK)	Placebo	Blind	3	April 30, 2018	Melanoma	RFS	OS
EORTC 1325-MG/KEYNOTE-054^7^	Pembrolizumab	MAB (anti–PD-L1 or anti– PD-1)	Placebo	Blind	3	February, 15 2019	Melanoma	RFS	OS
KATHERINE^8^	Trastuzumab emtansine	ADC (*ERBB2*)	Trastuzumab	Open	3	May 3, 2019	Breast	iDFS	OS, QLQ-C30, QLQ-BR23
ADAURA^5^	Osimertinib	Kinase inhibitor (EGFR)	Placebo	Blind	3	December 18, 2020	Non–small cell lung cancer	DFS	OS, SF-36
CheckMate 577^9^	Nivolumab	MAB (anti–PD-L1 or anti– PD-1)	Placebo	Blind	3	May 20, 2021	Esophagus or GEJ	DFS	OS
KEYNOTE-522^10^	Neoadjuvant pembrolizumab plus chemotherapy plus adjuvant pembrolizumab	MAB (anti–PD-L1 or anti– PD-1)	Placebo plus NAC chemotherapy	Blind	3	July 26, 2021	Breast	pCR and EFS	OS
CheckMate 274^11^	Nivolumab	MAB (anti–PD-L1 or anti– PD-1)	Placebo	Blind	3	August 19. 2021	Urothelial	DFS	OS
monarchE^18^	Abemaciclib	Kinase inhibitor (CDK4/6)	Standard of care	Open	3	October 12, 2021	Breast	iDFS	OS, FACIT-B, FACIT-F, and FACIT-ES
IMpower010^12^	Atezolizumab	MAB (anti–PD-L1 or anti– PD-1)	BSC	Open	3	October 15, 2021	Non–small cell lung cancer	DFS	OS
KEYNOTE-564^13^	Pembrolizumab	MAB (anti–PD-L1 or anti– PD-1)	Placebo	Blind	3	November 17, 2021	Renal cell	DFS	OS, QLQ-C30, FKSI-DRS
KEYNOTE-716^14^	Pembrolizumab	MAB (anti–PD-L1 or anti– PD-1)	Placebo	Blind	3	December 3, 2021	Melanoma	RFS	OS
CheckMate 816^15^	Neoadjuvant nivolumab plus platinum-based chemotherapy	MAB (anti–PD-L1 or anti– PD-1)	Platinum-based chemotherapy	Open	3	March 4, 2022	Non–small cell lung cancer	pCR plus EFS	OS
OlympiA^16^	Olaparib	Kinase inhibitor (PARP)	Placebo	Blind	3	March 11, 2022	Breast	iDFS	OS, QLQ-C30, FACIT-F
KEYNOTE-091/PEARLS^17^	Pembrolizumab	MAB (anti–PD-L1 or anti– PD-1)	Placebo	Blind	3	January 6, 2023	Non–small cell lung cancer	DFS	OS

Abbreviations: BSC, best supportive care; CDK4/6, cyclin-dependent kinase 4/6; DFS, disease-free survival; EFS, event-free survival; EGFR, epidermal growth factor receptor; FACIT-B, Functional Assessment of Cancer Therapy–Breast; FACIT-ES, Functional Assessment of Cancer Therapy–Endocrine Symptoms; FACIT-F, Functional Assessment of Cancer Therapy–Fatigue; FDA, Food and Drug Administration; FKSI-DRS, Functional Assessment of Cancer Therapy Kidney Cancer Symptom Index–Disease Related Symptoms; GEJ, gastroesophageal junction; iDFS, invasive disease-free survival; MAB, monoclonal antibody; NAC, neoadjuvant chemotherapy; OS, overall survival; pCR, pathologic complete response; PD-1, programmed cell death 1; PD-L1, programmed cell death ligand 1; Quality of Life Questionnaire–Breast Cancer, module 23; Quality of Life Questionnaire–Cancer, module 30; RFS, recurrence-free survival; SF-36, Short Form 36 Health Survey Questionnaire.

^a^ Adjuvant unless otherwise indicated.

Of 14 trials, 6 (43%)^8,10,11,16,17,18^ did not report any postrecurrence data. Of 8 trials with reported data,^5,6,7,9,12,13,14,15^ overall treatment was assessed as suboptimal in 6 (75%) (Table 2 and eResults 1 in Supplement 1). Among those 6 trials, in 1 trial,^14^ data were reported in aggregate and we could not exclude that the systemic treatment received at recurrence was optimal; however, the overall access to any therapy at recurrence was assessed as suboptimal. In the 2 trials (14%)^6,7^ coded with an overall optimal postrecurrence care, data were presented in aggregate; we could not rule out that treatment was optimal. The Figure shows the proportion of patients who received preferred treatments among patients who received any systemic therapy at recurrence in those trials. No trials used inappropriate crossover. Of the 2 trials^5,16^ with a significant OS benefit, 1 trial^5^ reported subpar postrecurrence data and the other^16^ did not report any postrecurrence data. Other postprogression and OS data are reported in Table 2.

**Table 2.** Included Registration Trials in the Neoadjuvant or Adjuvant Setting Between 2018 and 2023 With Postrecurrence Data

Trial name	Experimental arm^a^	Reported postprogression data	Standard of care systemic therapy at recurrence	Optimal access to any therapy on recurrence	Optimal overall assessment	Significant survival benefit
COMBI-AD^6^	Dabrafenib-trametinib	Yes	Yes	Yes	Yes	No
EORTC 1325-MG/KEYNOTE-054^7^	Pembrolizumab	Yes	Yes	Yes	Yes	No
KATHERINE^8^	Trastuzumab emtansine	No	NA	NA	NA	No
ADAURA^5^	Osimertinib	Yes	No	No	No	Yes
CheckMate 577^9^	Nivolumab	Yes	No	NA	No	No
KEYNOTE-522^10^	Neoadjuvant pembrolizumab plus chemotherapy plus adjuvant pembrolizumab	No	NA	NA	NA	No
CheckMate 274^11^	Nivolumab	No	NA	NA	NA	No
monarchE^18^	Abemaciclib	No	NA	NA	NA	No
IMpower010^12^	Atezolizumab	Yes	No	No	No	No
KEYNOTE-564^13^	Pembrolizumab	Yes	No	No	No	No
KEYNOTE-716^14^	Pembrolizumab	Yes	Yes	No	No	No
CheckMate 816^15^	Neoadjuvant nivolumab plus platinum-based chemotherapy	Yes	No	NA	No	No
OlympiA^16^	Olaparib	No	NA	NA	NA	Yes
KEYNOTE-091/PEARLS^17^	Pembrolizumab	No	NA	NA	NA	No

Abbreviation: NA, not assessable.

^a^ Adjuvant unless otherwise indicated.

**Figure.**
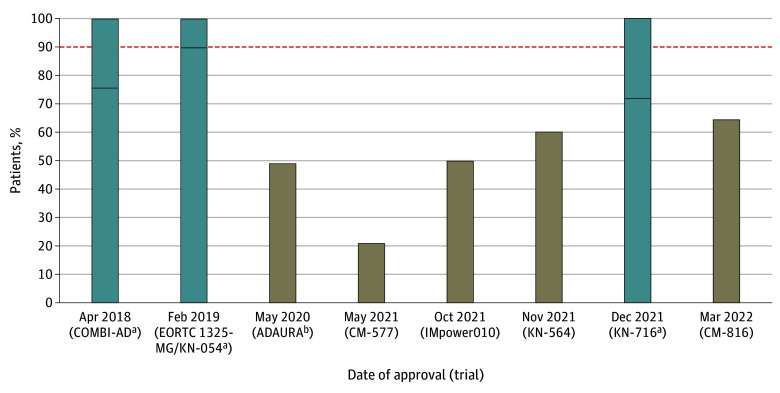
Proportion of Patients Who Received Standard of Care Among Patients Who Received Systemic Therapy at Disease Recurrence in 8 Trials With Postrecurrence Data Standard of care treatments (preferred treatments) used for each trial are detailed in the eResults 1 in Supplement 1. A detailed explanation of estimates is provided in the eMethods in Supplement 1. The dotted line corresponds to a prespecified rule that at least 90% of patients receiving systemic therapy at recurrence should receive an optimal therapy. CM indicates CheckMate; KN, KEYNOTE. ^a^Postrecurrence data were provided in aggregate; maximum and minimum access to optimal therapies (horizontal lines) was estimated. ^b^Maximum estimate.

## Discussion

This systematic review found that postrecurrence treatment was either not reported or reflected suboptimal care in most trials leading to an FDA approval of systemic therapies in the adjuvant or neoadjuvant setting between 2018 and 2023. This is consistent with a previous finding in advanced disease in which only 12% of FDA registration trials had available postprogression data assessed as optimal.^4^

To evaluate postprotocol therapy, we assessed 2 questions. One is whether control arm participants who received treatment at the time of relapse received the best available care. We found that most trials with assessable data reported subpar therapy. For example, in the ADAURA trial,^5^ 38.5% of patients who presented with a recurrence (excluding death) received osimertinib, which has been the standard of care since the FLAURA trial.^19^ Even when considering only patients receiving systemic therapy at relapse, only 48.8% received osimertinib (maximum estimate).^5^

Another question was related to the overall access to any subsequent therapy in both arms. In optimal care situations, a disease-free survival (DFS) benefit may be diluted by subsequent therapy.^20^ Conversely, in places where access to subsequent treatment is limited, a DFS benefit is more likely to translate into an OS benefit because fewer options are available after recurrence.

In the IMpower010 trial,^21^ patients with resected stage IB (≥4-cm tumor) to IIIA non–small cell lung cancer were randomized to adjuvant atezolizumab or best supportive care. Of patients presenting a recurrence in the control arm, 67% of them received a systematic treatment. However, in the general population, 77% of patients with stage II to IIIA disease who presented with a recurrence received a subsequent systemic therapy.^22^ In contrast to the lower percentage of people who received subsequent therapy in the IMpower010 trial than in the general population, we believe that this percentage should be higher in trials because stringent inclusion and exclusion criteria in trials select for patients with better health and fewer comorbidities.^23^ Clinical data as a benchmark can be suboptimal due to issues like poor generalizability; however, no trial in the present study was considered to be suboptimal based solely on this aspect.

In the 2 trials^5,16^ showing a significant OS benefit, none reported optimal postrecurrence care. However, postrecurrence data are vital in interpreting downstream end points like second progression-free survival or OS. While event-free survival or DFS have historically been considered surrogate markers for clinical efficacy, their direct clinical relevance as stand-alone end points remains under debate.^24,25^ For patients who value their time without treatment or who value a survival benefit, positive OS results may be poorly informed in the context of suboptimal postrelapse therapy. Because a small proportion of patients receiving perioperative therapy ultimately benefit, discussing treatment goals with patients is critical.^1^

Our findings add to other issues like high costs^26^ and the risk of bias in quality-of-life results.^27^ Regulators could restrict enrollment to countries with optimal care access. However, this would impair the diverse representation of patients in trials and deepen inequality regarding access to innovation worldwide. Alternatively, they could mandate sponsor-funded crossover at recurrence when the best available therapy is known. We also propose illustrations of postrecurrence data, when available, to aid in shared decision-making (eFigure 2 in Supplement 1).

### Strengths and Limitations

This study has strengths. First, to our knowledge, it is the first comprehensive evaluation of postrecurrence data in the perioperative settings of cancer treatment. Second, our method has been used in other contexts (advanced and metastatic settings), ensuring consistency. This study also has limitations. We did not have access to individual patient data, thus limiting a full assessment of postprogression data; however, our conservative categorization leaned toward overestimating the quality of postrecurrence therapies rather than underestimating it.

## Conclusions

This systematic review found that 43% of randomized clinical trials of anticancer treatment in the adjuvant or neoadjuvant context failed to present any assessable postrecurrence treatment data. In instances in which these data were shared, postrecurrence treatment was suboptimal 75% of the time. The findings suggest that regulatory bodies should enforce rules stipulating that patients have access to the best standard of care at recurrence in global trials.

## Notes

Supplement 1.
eMethods.
**eResults 1.** Therapies Considered as Preferred Options Upon Recurrence in Trials With Assessable Data With References (Rule 1, n = 8) **eResults 2.** Real-World Data References for Trials Assessed With Suboptimal Access to Any Postrecurrence Therapy (Rule 3, n = 4) **eResults 3.** Included Trials With NCT Numbers and References. **eFigure 1.** Flowchart of the Approval Selection Process and Reasons for Exclusion. **eFigure 2.** Trials in the Adjuvant Setting With Overall Survival Benefit (N = 2), With Key Postrecurrence Data (When Available)
eReferences.

Supplement 2.
Data Sharing Statement
