# Supplementary material for: Postrecurrence Treatment in Neoadjuvant or Adjuvant FDA Registration Trials: A Systematic Review
Source: JAMA Oncol. 2024 Jun 20;10(8):1055–9. doi: 10.1001/jamaoncol.2024.1569 (PMC11190827; doi:10.1001/jamaoncol.2024.1569)
Supplement: Supplement 1. — eMethods. eResults 1. Therapies Considered as Preferred Options Upon Recurrence in Trials With Assessable Data With References (Rule 1, n = 8) eResults 2. Real-World Data References for Trials Assessed With Suboptimal Access to Any Postrecurrence Therapy (Rule 3, n = 4) eResults 3. Included Trials With NCT Numbers and References. eFigure 1. Flowchart of the Approval Selection Process and Reasons for Exclusion. eFigure 2. Trials in the Adjuvant Setting With Overall Survival Benefit (N = 2), With Key Postrecurrence Data (When Available) eReferences. [file jamaoncol-e241569-s001.pdf]

Supplementary Online Content

Olivier T, Haslam A, Prasad V. Postrecurrence treatment in neoadjuvant or adjuvant FDA registration trials: a systematic review. *JAMA Oncol*. Published online June 20, 2024.
doi:10.1001/jamaoncol.2024.1569

eMethods.
eResults 1. Therapies Considered as Preferred Options Upon Recurrence in Trials With Assessable Data With References (Rule 1, n = 8)
eResults 2. Real-World Data References for Trials Assessed With Suboptimal Access to Any Postrecurrence Therapy (Rule 3, n = 4)
eResults 3. Included Trials With NCT Numbers and References.
eFigure 1. Flowchart of the Approval Selection Process and Reasons for Exclusion.
eFigure 2. Trials in the Adjuvant Setting With Overall Survival Benefit (N = 2), With Key Postrecurrence Data (When Available)
eReferences.

This supplementary material has been provided by the authors to give readers additional information about their work.

© 2024 Olivier T et al. *JAMA Oncology.*

eMethods:

Data Abstraction

Information abstracted for each approval comprised the date of approval; approval's type (regular or accelerated); trial's name; NCT identifier, tumor type; experimental arm and its mechanism of action; control arm; setting (adjuvant, neoadjuvant, or both); design (open or blinded); phase; sponsor; primary endpoint; whether overall survival was a primary endpoint (yes or no); post-recurrence data; whether overall survival (OS) results were reported (yes or no); the hazard ratio, confidence intervals, and p values of OS and if the results were considered statistically significant (yes or no).

We abstracted whether post-recurrence data were available (yes or no). We abstracted, when present, in the control arm: the total number of patients included; the number of patients having an event-free survival event (recurrence-free survival, relapse-free survival, event-free survival, disease-free survival (DFS), invasive DFS); the number of patients receiving any subsequent treatment; the number of patients receiving any systemic subsequent treatment; and the number of patients receiving appropriate subsequent systemic therapy. Among patients having recurrence in the control arm, we calculated the proportion of those receiving any therapy; those receiving any systemic therapy; and those receiving appropriate systemic therapy.

For each trial, we look at FDA reviews available on the FDA website, published data, and data from meeting abstracts. We retained the most mature data for analysis. Because the number of events is central in assessing the quality of post-recurrence treatment, we used the number of events reported in the same publication of the abstracted post-recurrence data. In some instances, we used another publication for overall survival results, even where no post-recurrence data were presented in this publication. Publications and meeting abstracts were identified using the publications referred on clinicaltrials.gov, and we also used Google Scholar to look for meeting abstracts that may not be indexed in clinicaltrials.gov.

Two of the authors (AH, TO) independently reviewed and abstracted data from each article. A third reviewer (VP) adjudicated any discrepancies.

Post-recurrence assessment

Post-recurrence therapy is the counterpart, in the neoadjuvant and adjuvant settings, of post-progression therapy in the advanced or metastatic settings. We used the terms "post-recurrence" therapy to define the treatment given upon disease recurrence. This encompassed "crossover" and "post-protocol", which may be used interchangeably in publications.

Post-recurrence data may be reported in aggregate with same patients possibly receiving different lines of therapies. It may therefore be challenging to assess how many patients got access to optimal care, particularly when the preferred systemic therapy is not unique. In those cases, we estimated two scenarios. For instance, in an adjuvant melanoma trial, optimal therapies upon progression are BRAF and MEK inhibitors or anti-PD1 containing regimens. In a first scenario, we selected the highest number of patients receiving one of the two standard-of-care strategies, and made the "worst case scenario" assumption, assuming the same patients also received the other standard-of-care therapy as another line of therapy, and the remaining patients had no access to either option. Conversely, we estimated a "best case scenario", adding up the numbers of patients receiving different therapies, which often resulted in more than 100% of patients.
In other trials, the number of patients undergoing systemic therapy may not be specifically reported. We then estimated this metric based on the most conservative assumption. In ADAURA, this number was estimated by the number of patients receiving any EGFR-TKI (n = 162), even if the real number was likely higher.

The duration of follow-up logically affects post-recurrence data, with more patients relapsing with a longer follow-up. However, we limited its influence on our assessment by focusing only on therapies received in patients who relapsed.

Two independent reviewers (TO and VP) evaluated the post-recurrence data of trials, and their final decision was based on mutual agreement. Each rule was assessed in each trial. If a trial did not satisfy at least one rule, it was determined that post-recurrence care was subpar. When a lack of granular data precluded a definite ascertainment, we coded the trial as having optimal post-recurrence therapy.

© 2024 Olivier T et al. *JAMA Oncology.*

eResults 1: Therapies Considered As Preferred Options Upon Recurrence In Trials With Assessable Data With References (rule 1, n=8)

COMBI-AD: Checkpoint inhibitor(s) or BRAF plus MEK inhibitors.[1,2,3]
EORTC 1325-MG/KEYNOTE-054: Checkpoint inhibitor(s) or BRAF plus MEK inhibitors.[1,2,3]
ADAURA: Osimertinib.[4]
CheckMate 577: Checkpoint inhibitor containing regimen.[5,6]
Impower010: Checkpoint inhibitor containing regimen.[7,8]
KEYNOTE-564: Checkpoint inhibitor containing regimen.[9,10,11]
KEYNOTE-716: Checkpoint inhibitor(s) or BRAF plus MEK inhibitors.[1,2,3]
CheckMate-816: Checkpoint inhibitor containing regimen.[7,8]

eResults 2: Real-World Data References For Trials Assessed with Suboptimal Access to Any Post-Recurrence Therapy

ADAURA: Sara Curuvilla et al. 2023.[12]
Impower010: Cortinovis et al. 2023.[13]
KEYNOTE-564: Dabestani et al. 2019.[14]
KEYNOTE-716: O'Sullivan et al. 2023.[15]

eResults 3: Included Trials With NCT Numbers And References.

COMBI-AD, NCT01682083[16]
EORTC 1325-MG/KEYNOTE-05, NCT02362594[17]
KATHERINE, NCT01772472[18]
ADAURA, NCT02511106[19]
CheckMate 577, NCT02743494[20]
KEYNOTE-522, NCT03036488[21]
CheckMate 274, NCT02632409[22]
IMpower010, NCT02486718[23]
KEYNOTE-564, NCT03142334[24]
KEYNOTE-716, NCT03553836[25]
CheckMate 816, NCT02998528[26]
OlympiA, NCT02032823[27]
KEYNOTE-091/PEARLS, NCT02504372[28]
monarchE, NCT03155997[29]

© 2024 Olivier T et al. *JAMA Oncology.*

eFigure 1: Flowchart Of The Approval Selection Process And Reasons For Exclusion.

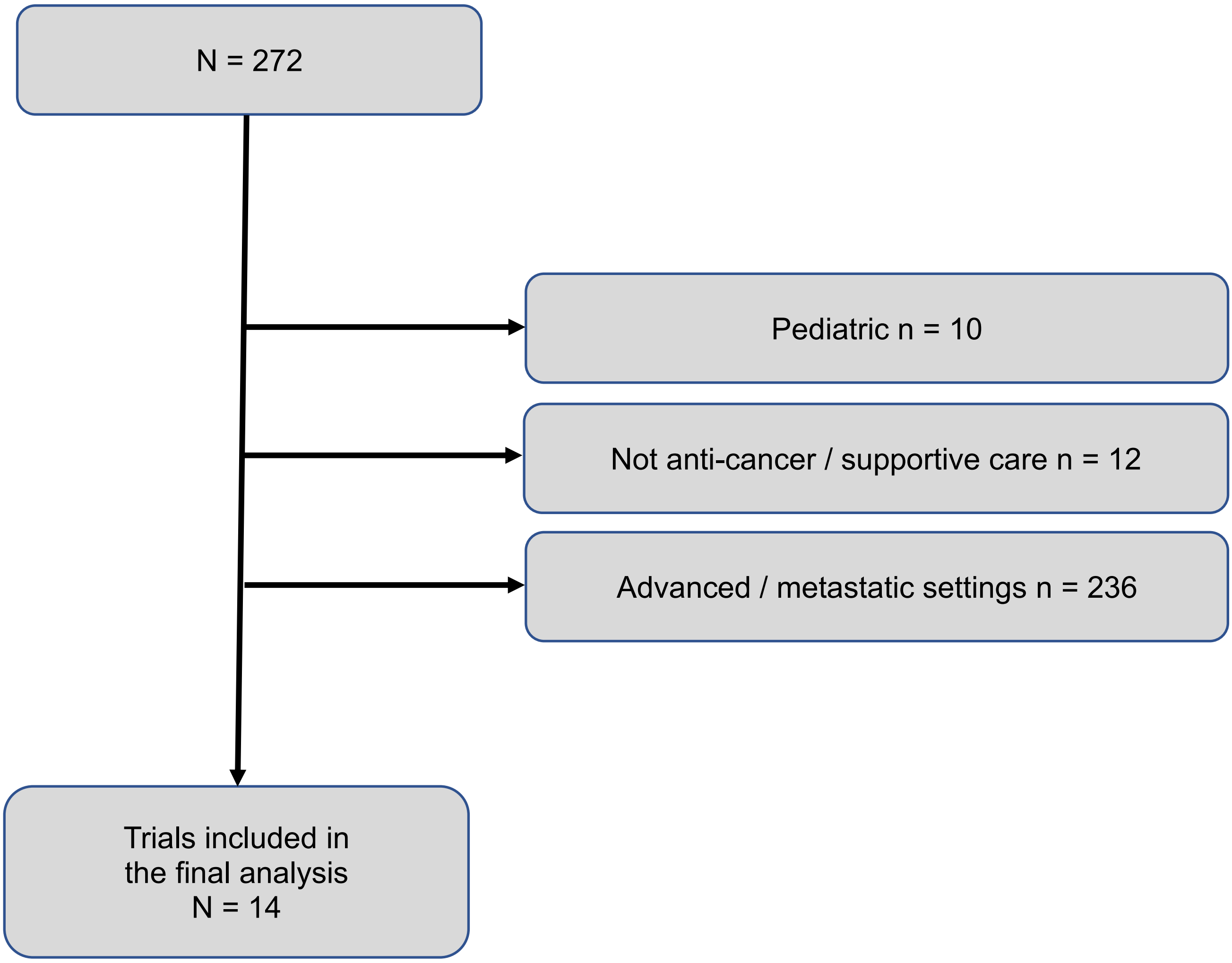

© 2024 Olivier T et al. *JAMA Oncology.*

eFigure 2: Trials In The Adjuvant Setting With Overall Survival Benefit (N = 2), With Key Post-recurrence Data (When Available)

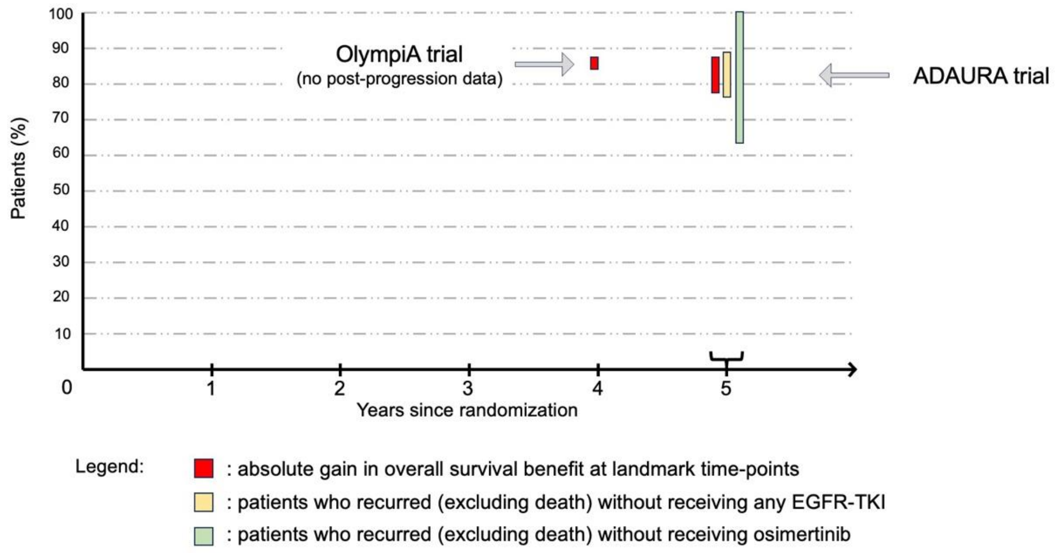

© 2024 Olivier T et al. *JAMA Oncology.*

7 - References:

1. Wolchok JD, Chiarion-Sileni V, Gonzalez R, Grob JJ, Rutkowski P, Lao CD, et al. Long-Term Outcomes With Nivolumab Plus Ipilimumab or Nivolumab Alone Versus Ipilimumab in Patients With Advanced Melanoma. JCO. 2022 Jan 10;40(2):127–37.
2. Ascierto PA, McArthur GA, Dréno B, Atkinson V, Liszkay G, Giacomo AMD, et al. Cobimetinib combined with vemurafenib in advanced BRAFV600-mutant melanoma (coBRIM): updated efficacy results from a randomised, double-blind, phase 3 trial. The Lancet Oncology. 2016 Sep 1;17(9):1248–60.
3. Robert C, Karaszewska B, Schachter J, Rutkowski P, Mackiewicz A, Stroiakovski D, et al. Improved Overall Survival in Melanoma with Combined Dabrafenib and Trametinib. New England Journal of Medicine. 2015 Jan 1;372(1):30–9.
4. Soria JC, Ohe Y, Vansteenkiste J, Reungwetwattana T, Chewaskulyong B, Lee KH, et al. Osimertinib in Untreated EGFR-Mutated Advanced Non–Small-Cell Lung Cancer. New England Journal of Medicine. 2018 Jan 11;378(2):113–25.
5. Doki Y, Ajani JA, Kato K, Xu J, Wyrwicz L, Motoyama S, et al. Nivolumab Combination Therapy in Advanced Esophageal Squamous-Cell Carcinoma. New England Journal of Medicine. 2022 Feb 3;386(5):449–62.
6. Kato K, Cho BC, Takahashi M, Okada M, Lin CY, Chin K, et al. Nivolumab versus chemotherapy in patients with advanced oesophageal squamous cell carcinoma refractory or intolerant to previous chemotherapy (ATTRACTION-3): a multicentre, randomised, open-label, phase 3 trial. The Lancet Oncology. 2019 Nov 1;20(11):1506–17.
7. Gandhi L, Rodríguez-Abreu D, Gadgeel S, Esteban E, Felip E, De Angelis F, et al. Pembrolizumab plus Chemotherapy in Metastatic Non–Small-Cell Lung Cancer. New England Journal of Medicine. 2018 May 31;378(22):2078–92.
8. Paz-Ares L, Luft A, Vicente D, Tafreshi A, Gümüş M, Mazières J, et al. Pembrolizumab plus Chemotherapy for Squamous Non–Small-Cell Lung Cancer. New England Journal of Medicine. 2018 Nov 22;379(21):2040–51.
9. Rini BI, Plimack ER, Stus V, Gafanov R, Hawkins R, Nosov D, et al. Pembrolizumab plus Axitinib versus Sunitinib for Advanced Renal-Cell Carcinoma. N Engl J Med. 2019 Mar 21;380(12):1116–27.
10. Motzer RJ, Escudier B, McDermott DF, George S, Hammers HJ, Srinivas S, et al. Nivolumab versus Everolimus in Advanced Renal-Cell Carcinoma. New England Journal of Medicine. 2015 Nov 5;373(19):1803–13.
11. Meirson T, Neiman V, Sternschuss M, Markel G, Tannock IF. Clarification needed for pembrolizumab as adjuvant therapy in clear cell renal cell carcinoma. Lancet Oncol. 2022 Nov;23(11):e489.
12. Sara Kuruvilla M, Liu G, Syed I, Gwadry-Sridhar F, Sheffield BS, Sachdeva R, et al. EGFR mutation prevalence, real-world treatment patterns, and outcomes among patients with resected, early-stage, non-small cell lung cancer in Canada. Lung Cancer. 2022 Nov;173:58–66.
13. Cortinovis DL, Perrone V, Giacomini E, Sangiorgi D, Andretta M, Bartolini F, et al. Epidemiology, Patients' Journey and Healthcare Costs in Early-Stage Non-Small-Cell Lung Carcinoma: A Real-World Evidence Analysis in Italy. Pharmaceuticals (Basel). 2023 Feb 27;16(3):363.
14. Dabestani S, Beisland C, Stewart GD, Bensalah K, Gudmundsson E, Lam TB, et al. Long-term Outcomes of Follow-up for Initially Localised Clear Cell Renal Cell Carcinoma: RECUR Database Analysis. European Urology Focus. 2019 Sep 1;5(5):857–66.
15. O'Sullivan DE, Boyne DJ, Gogna P, Brenner DR, Cheung WY. Understanding Real-World Treatment Patterns and Clinical Outcomes among Metastatic Melanoma Patients in Alberta, Canada. Curr Oncol. 2023 Apr 13;30(4):4166–76.
16. Dummer R, Hauschild A, Santinami M, Atkinson V, Mandalà M, Kirkwood JM, et al. Five-Year Analysis of Adjuvant Dabrafenib plus Trametinib in Stage III Melanoma. New England Journal of Medicine. 2020 Sep 17;383(12):1139–48.
17. Eggermont AMM, Kicinski M, Blank CU, Mandala M, Long GV, Atkinson V, et al. Five-Year Analysis of Adjuvant Pembrolizumab or Placebo in Stage III Melanoma. NEJM Evidence. 2022 Oct 25;1(11):EVIDoa2200214.
18. von Minckwitz G, Huang CS, Mano MS, Loibl S, Mamounas EP, Untch M, et al. Trastuzumab Emtansine for Residual Invasive HER2-Positive Breast Cancer. New England Journal of Medicine. 2019 Feb 14;380(7):617–28.
19. Tsuboi M, Herbst RS, John T, Kato T, Majem M, Grohé C, et al. Overall Survival with Osimertinib in Resected EGFR-Mutated NSCLC. New England Journal of Medicine. 2023 Jul 13;389(2):137–47.
20. Kelly RJ, Ajani JA, Kuzdzal J, Zander T, Van Cutsem E, Piessen G, et al. Adjuvant Nivolumab in Resected Esophageal or Gastroesophageal Junction Cancer. New England Journal of Medicine. 2021 Apr

© 2024 Olivier T et al. *JAMA Oncology.*

1;384(13):1191–203.
21. Schmid P, Cortes J, Dent R, Pusztai L, McArthur H, Kümmel S, et al. Event-free Survival with Pembrolizumab in Early Triple-Negative Breast Cancer. New England Journal of Medicine. 2022 Feb 10;386(6):556–67.
22. Bajorin DF, Witjes JA, Gschwend JE, Schenker M, Valderrama BP, Tomita Y, et al. Adjuvant Nivolumab versus Placebo in Muscle-Invasive Urothelial Carcinoma. New England Journal of Medicine. 2021 Jun 3;384(22):2102–14.
23. Felip E, Altorki N, Zhou C, Csőszi T, Vynnychenko I, Goloborodko O, et al. Adjuvant atezolizumab after adjuvant chemotherapy in resected stage IB–IIIA non-small-cell lung cancer (IMpower010): a randomised, multicentre, open-label, phase 3 trial. The Lancet. 2021 Oct 9;398(10308):1344–57.
24. Powles T, Tomczak P, Park SH, Venugopal B, Ferguson T, Symeonides SN, et al. Pembrolizumab versus placebo as post-nephrectomy adjuvant therapy for clear cell renal cell carcinoma (KEYNOTE-564): 30-month follow-up analysis of a multicentre, randomised, double-blind, placebo-controlled, phase 3 trial. The Lancet Oncology. 2022 Sep 1;23(9):1133–44.
25. Luke JJ, Rutkowski P, Queirolo P, Vecchio MD, Mackiewicz J, Chiarion-Sileni V, et al. Pembrolizumab versus placebo as adjuvant therapy in completely resected stage IIB or IIC melanoma (KEYNOTE-716): a randomised, double-blind, phase 3 trial. The Lancet. 2022 Apr 30;399(10336):1718–29.
26. Forde PM, Spicer J, Lu S, Provencio M, Mitsudomi T, Awad MM, et al. Neoadjuvant Nivolumab plus Chemotherapy in Resectable Lung Cancer. New England Journal of Medicine. 2022 May 26;386(21):1973–85.
27. Geyer CE, Garber JE, Gelber RD, Yothers G, Taboada M, Ross L, et al. Overall survival in the OlympiA phase III trial of adjuvant olaparib in patients with germline pathogenic variants in BRCA1/2 and high-risk, early breast cancer. Annals of Oncology. 2022 Dec 1;33(12):1250–68.
28. O'Brien M, Paz-Ares L, Marreaud S, Dafni U, Oselin K, Havel L, et al. Pembrolizumab versus placebo as adjuvant therapy for completely resected stage IB–IIIA non-small-cell lung cancer (PEARLS/KEYNOTE-091): an interim analysis of a randomised, triple-blind, phase 3 trial. The Lancet Oncology. 2022 Oct 1;23(10):1274–86.
29. Johnston SRD, Toi M, O'Shaughnessy J, Rastogi P, Campone M, Neven P, et al. Abemaciclib plus endocrine therapy for hormone receptor-positive, HER2-negative, node-positive, high-risk early breast cancer (monarchE): results from a preplanned interim analysis of a randomised, open-label, phase 3 trial. The Lancet Oncology. 2023 Jan 1;24(1):77–90.

© 2024 Olivier T et al. *JAMA Oncology.*
