# Supplementary material for: Postrecurrence Treatment in Neoadjuvant or Adjuvant FDA Registration Trials: A Systematic Review
Source: JAMA Oncol. 2024 Jun 20;10(8):1055–9. doi: 10.1001/jamaoncol.2024.1569 (PMC11190827; doi:10.1001/jamaoncol.2024.1569)
Supplement: Supplement 2. — Data Sharing Statement [file jamaoncol-e241569-s002.pdf]

Data Sharing Statement

Olivier. Postrecurrence Treatment in Neoadjuvant or Adjuvant FDA Registration Trials. *JAMA Oncol*. Published June 20, 2024. doi:10.1001/jamaoncol.2024.1569

Data
Data available: Yes
Data types: Data (not involving human participants)
How to access data: All data on which this work was based are publicly available. The data generated during the study are available upon reasonable request from the corresponding author.
When available: With publication

Supporting Documents
Document types: Other (please specify)
Additional Information: All data on which this work was based are publicly available. The data generated during the study are available upon reasonable request from the corresponding author.
How to access documents: timothee.olivier@hcuge.ch
When available: With publication

Additional Information
Who can access the data: All data on which this work was based are publicly available. The data generated during the study are available upon reasonable request from the corresponding author.
Types of analyses: Any purpose.
Mechanisms of data availability: The data generated during the study are available upon reasonable request from the corresponding author.
